# Oral glucocorticoids and the risk of psychiatric and suicidal behaviour outcomes: a population-based cohort study

**DOI:** 10.1192/bjp.2025.128

**Published:** 2025-06-25

**Authors:** Tyra Lagerberg, Tapio T Gustafsson, Yasmina Molero, Julian Forton, Amir Sariaslan, Zheng Chang, Henrik Larsson, Paul Lichtenstein, Seena Fazel

**Affiliations:** 1 Department of Psychiatry, Warneford Hospital, University of Oxford, Oxford, United Kingdom; 2 Department of Medical Epidemiology and Biostatistics, Karolinska Institutet, Stockholm, Sweden; 3 Department of Forensic Psychiatry, University of Eastern Finland, Niuvanniemi Hospital, Kuopio, Finland; 4 Department of Clinical Neuroscience, Karolinska Institutet, Stockholm, Sweden; 5 The Children’s Hospital for Wales, Cardiff, United Kingdom; 6 Cardiff University School of Medicine, Cardiff, United Kingdom; 7 School of Medical Sciences, Örebro University, Örebro, Sweden; 8 Oxford Health NHS Foundation Trust

**Keywords:** glucocorticoid, corticosteroid, pharmacoepidemiology, psychiatric outcome, suicidal behaviour, self-controlled design, cohort study, population registers

## Abstract

**Background:**

Despite evidence of associations between glucocorticoid treatment and adverse psychiatric and suicidal behaviour outcomes, large-scale observational evidence for severe outcomes is lacking.

**Aim:**

To assess the risk of psychiatric and suicidal behaviour outcomes from glucocorticoid treatment using outcomes defined in specialist psychiatric care.

**Method:**

Using Swedish population registers, we identified 1,105,964 individuals ages 15-54 years that collected a glucocorticoid prescription in oral form 2006 to 2020. We investigated associations with a range of psychiatric outcomes: specialist healthcare contacts due to depressive, bipolar, anxiety, or schizophrenia-spectrum disorders; and deaths by suicide or specialist healthcare contacts due to suicide attempts (“suicidal behaviour”). We estimated hazard ratios (HRs) from Cox Proportional Hazards models in a medication-only cohort by comparing outcome rates during and outside treated periods within individuals. We further identified individuals with an autoimmune or gastrointestinal autoimmune disorder diagnosis and compared hazards of the outcomes between those who did and did not initiate a glucocorticoid.

**Results:**

We found increased hazards for the psychiatric outcomes, with HRs ranging from 1.08 (95% CI=1.00, 1.16) for depressive disorders, to 1.25 (95% CI=1.20, 1.31) for anxiety disorders. We found no clear association with suicidal behaviour (HR=1.06, 95% CI=0.96, 1.17). These findings were similar when stratified by age and sex. Within-individual associations were attenuated in those diagnosed with an autoimmune disorder or a gastrointestinal autoimmune disorder. The risk of anxiety and bipolar disorder outcomes appeared particularly elevated in the first weeks of treatment. Absolute rates were modestly elevated during treatment, and higher in those with a history of psychiatric disorders.

**Conclusions:**

Glucocorticoid treatment is associated with elevated risks of psychiatric specialist care contacts for a range of disorders. Individuals with psychiatric histories may require additional monitoring for psychiatric outcomes during glucocorticoid treatment.

## Introduction

Oral glucocorticoids, a type of corticosteroid, are immunosuppressant and anti-inflammatory agents (1) used for a wide range of indications, including autoimmune disorders (2). Despite their efficacy, there is concern about adverse neuropsychiatric and behavioural effects. These include acute outcomes such as severe anxiety attacks, mania, suicidal behaviour, and psychotic episodes (1). In support, a large cohort study in primary care data found that glucocorticoid treatment was associated with substantially elevated risks of suicidal behaviour, mania, and panic disorder over three months, and that a past history of mental health disorders increased the risk of the outcomes (3). This and other studies find that higher doses of glucocorticoids are associated with greater risks of psychiatric and behavioural outcomes (3–5), and that age is a moderator of the risks (3, 4). Prior studies have found varying impact of a history of psychiatric disorders (3, 6–8).

However, there is limited large-scale observational evidence considering a broad set of psychiatric outcomes defined by psychiatric healthcare admissions that likely represent more severe events. Further, we are not aware of studies that have used self-controlled designs, which account for all measured and unmeasured time-invariant confounding within individuals, including genetic make-up and childhood environment; or of studies that have employed a target trial approach to ensure a range of common biases are mitigated and that a clear clinical question is formulated (9).

In this study, we therefore aimed to explore the association between glucocorticoid treatment and psychiatric and suicidal outcomes as defined by specialist care contacts in a nationally representative data linkage using two complementary designs. First, we considered all those ever dispensed a glucocorticoid (medication-only cohort) and used a self-controlled design to account for factors that remain stable in individuals. We stratified these analyses by age, whether individuals had a history of psychiatric diagnoses, and by receipt of an autoimmune disorder diagnosis or a gastrointestinal autoimmune disorder diagnosis. We also considered the impact of duration of treatment. Second, we followed individuals from their first diagnosis of an autoimmune disorder or a gastrointestinal autoimmune disorder (indication cohorts) and compared psychiatric risks in those who did and did not initiate a glucocorticoid medication using a target trial approach. These analyses were also stratified on a history of psychiatric disorders.

## Methods and Materials

### Ethics and consent

Ethical approval was secured from the Stockholm Regional Ethics Committee (Stockholm, Sweden, reference numbers 2013/862-31/5 and 2020-06540). The need for informed consent was waived according to Swedish law, on the basis that the research was register-based and data was pseudonymised.

### Data sources

Information was linked across Swedish national registers using unique personal identification numbers (10). We extracted prescription information from the Swedish Prescribed Drug Register, where dispensed (prescribed and collected) pharmaceuticals since 2005 are documented (11); information on inpatient and specialist outpatient care from the National Patient Register, which documents this information since 1973 (for inpatient care) and 2001 (for specialist outpatient care) (12); demographic data from the Total Population Register (13); and emigration data from the Migration Register (13). Causes and dates of death were extracted from The Cause of Death Register (14).

### Cohort

#### Medication-only cohort

We identified individuals aged 15-54 years that collected at least one glucocorticoid prescription (see Measures section for details on ATC codes) in oral form during 1 January 2006 to 31 December 2020. We focused on individuals aged below 54 to estimate effects in adults. We excluded individuals who were ever dispensed a glucocorticoid where the prescription text indicated they had Addison’s disease, given that steroid treatment in these individuals is intended to replace insufficient endogenous production. Follow-up started 1 January 2006, or on the date of the individual’s 15^th^ birthday, whichever came last. It ended at date of first emigration; date of death; date an individual reached age 55; or 31 December 2020, whichever occurred first.

#### Indication cohorts – target trial emulation

As an alternative to the medication-only cohort, we defined indication cohorts in order to emulate two target trials. This allowed us to define clear clinical questions – that is, what is the effect of glucocorticoid treatment following a) any autoimmune diagnosis, and b) a gastrointestinal autoimmune diagnosis on psychiatric and suicidal behaviour risks? See Table S1 for a description of the target trials and how we emulated them.

We identified two cohorts based on common disorders indicating glucocorticoid treatment – any autoimmune disorders and gastrointestinal autoimmune disorders. See Table S2 for the diagnoses considered, defined using the International Classification of Diseases, 10^th^ revision (ICD-10) codes (15). We selected individuals who received a diagnosis of either of these disorders between 1 January 2006 and 30 November 2019, choosing the first recorded diagnosis as index. We then excluded individuals who had been prescribed a glucocorticoid less than 180 days before they received their diagnosis to ensure that all included individuals had a washout period from glucocorticoid treatment of at least 180 days. Anyone initiating a glucocorticoid within 28 days of their diagnosis was assigned as an initiator, and anyone who did not as a control. The start of follow-up was the date of glucocorticoid dispensation among initiators. Among controls, the time between the diagnosis date and dispensation date in initiators was randomly assigned to define the follow-up start (16). We carried out intention-to-treat and per-protocol analyses (see Analyses section). For intention-to-treat analyses, follow-up ended at 365 days after follow-start. For per-protocol analyses, follow-up ended at 365 days after follow-start unless individuals stopped adhering to their baseline treatment strategy, at which point they were censored.

### Measures

#### Exposure

We considered any glucocorticoid medication licensed for sale in Sweden during the study period. We did not have information on individual Anatomical Therapeutic Chemical (ATC) codes below the second level (H02) for the main cohort, so we could not directly select only glucocorticoid dispensations (H02AB). However, the only medication in the H02 category that is not a glucocorticoid and licensed for sale in Sweden is fludrocortisone (H02AA02), a mineralocorticoid used for Addison’s disease and congenital adrenal hyperplasia. Both are rare. An estimated 1,300 Swedish residents have Addison’s disease (17) and we excluded any individual where their prescription text indicated this disease, though we did not have structured diagnostic information on Addison’s disease. Meanwhile, only 606 individuals born 1915-2011 had congenital adrenal hyperplasia (18). For completeness, we carried out sensitivity analyses in a subset of the follow-up (2006-2013) where we were able to restrict to only glucocorticoid prescriptions (H02AB, see Analyses section). We created continuous treatment periods on the assumption that any prescriptions falling within 120 days of each other within an individual belonged to the same treatment period (19). The treatment period started at the date of the first prescription in the period. For the last or single prescription in a treatment period, 14 days were added to the end to define the date of treatment period end (19). Continuous treatment periods were considered in the analyses of the medication-only cohort and in per-protocol analyses in the indication cohorts.

#### Outcomes

We considered any unplanned specialist psychiatric healthcare contact where diagnoses were made for anxiety (F4), bipolar (F25.0, F30, F31, F34.0), depressive (F32-F33, F34 excluding F34.0, F38-F39), or schizophrenia-spectrum disorders (ICD-10 codes: F2, excluding F25.0) (20). For suicidal behaviour, we considered any unplanned healthcare contact where the diagnosis was suicidal attempt, or death by suicide; of either known (X60-X84) or unknown (Y10-Y34) intent. The date of patient admission to care was assumed to be the date of the event.

#### Covariates

In the medication-only cohort, all analyses were adjusted for age. Between-individual analyses were additionally adjusted for sex. In the indication cohorts, all analyses were adjusted for sex, age, year of follow-up start, highest level of attained education of individual and their parents, highest income category between individual and parents, and diagnoses at baseline (anxiety, bipolar disorder, depression, psychotic disorder, history of suicide attempt). The only variable with missing information was highest attained education between individuals and their parents, where missingness was between 1% and 2% (Table S3, Table S4). Missing values for education were treated as a separate category in the education variable.

### Analyses

#### Medication-only cohort

We used stratified Cox proportional hazards models to estimate within-individual Hazard Ratios (HRs). We compared the hazards of the outcomes during treated and untreated times within the same individual, ensuring that all time-invariant confounding was controlled for (21, 22). Analyses were carried out overall; and stratified by: sex, age category (15-24 years, 25-34 years, 35-44 years, and 45-54 years), and a history of any of the outcomes at start of follow-up (past mental health disorder). We also conducted a sensitivity analysis excluding any individual ever prescribed an inhaled glucocorticoid (ATC code R03BA). All analyses were adjusted for time-varying age.

To investigate whether there were periods of high or low risk during glucocorticoid treatment, we carried out analyses looking at the outcome in each of the periods since treatment start: <14 days, 14-119 days, 120-364 days, and >364 days, using any off-treatment period as the reference. We also considered average daily dose of glucocorticoid medication collected during treatment. We calculated this by taking the sum of the amount of medication – expressed as defined daily dose (DDD) – that was dispensed over follow-up. We then divided the cumulative DDD by the length of treatment in days. We classified <0.5 DDDs per day as low dose, 0.5-1.5 DDDs per day as medium dose, and >1.5 DDDs per day as high dose (23).

We further stratified by diagnosis of an autoimmune disorder or a gastrointestinal autoimmune disorder. These diagnoses were defined as being present after the first recorded diagnosis from 1 January 2001 to 31 December 2020. To investigate the impact of between-individual confounding in the medication-only cohort, we carried out between-individual analyses using Cox Proportional Hazards models – these were adjusted for age and sex. We used robust sandwich covariance when calculating the confidence intervals to account for correlation of person-time within individuals (24).

Finally, to assess whether our results were impacted by the potential inclusion of non-glucocorticoid corticosteroids, we restricted analyses to the follow-up period of 1 January 2006 to 31 December 2013. In this data, we had information to the 5^th^ ATC level (e.g. H02AB01), so were able to ensure that the cohort contained only dispensations of glucocorticoids.

#### Indication cohorts

We used pooled logistic regression models with product terms between treatment and time (25) to estimate cumulative incidence and HRs for each of the outcomes at 14 days, 120 days, and 365 days of follow-up. We accounted for baseline confounders by applying inverse probability weighting (IPW) (26), and calculated the standardized mean difference (SMD) between confounder distributions in initiators and controls before and after weighting. We conducted both intention-to-treat and per-protocol analyses (Table S1). In intention-to-treat analyses, individuals were assumed to adhere to their assigned baseline treatment throughout follow-up. In per-protocol analyses, we censored individuals when they stopped adhering to the treatment strategy they were assigned at baseline. For initiators, this occurred if the individual terminated their treatment during follow-up. For controls, this occurred if the individual initiated glucocorticoid treatment during follow-up. We estimated time-varying treatment adherence weights using baseline confounders and specialist outpatient or inpatient healthcare contacts over follow-up. We weighted each two-week period of follow-up by the product of the time-varying IPW weights and baseline IPW weights. Weights were stabilized and truncated at the 99th percentile. We used non-parametric bootstraps over 500 samples to estimate 95% confidence intervals. All analyses were run overall, and intention-to-treat analyses were stratified by a history of the outcomes at start of follow-up (past mental health disorder).

## Results

We identified 1,105,964 individuals who had at least one dispensed glucocorticoid prescription between 1 January 2006 and 31 December 2020 at ages 15-54 years (Table 1). Of those prescribed glucocorticoids, 642,567 (58.1%) were female. Mean length of follow-up was around 12 years in the medication-only cohort. During follow-up, there were unplanned specialist healthcare contacts for an anxiety disorder in 14.1% of the cohort, bipolar disorders (2.6%), depressive disorders (7.0%), schizophrenia-spectrum disorders (2%), and suicidal behaviour (4.1%). These proportions were similar between men and women.

**Figure 1:**
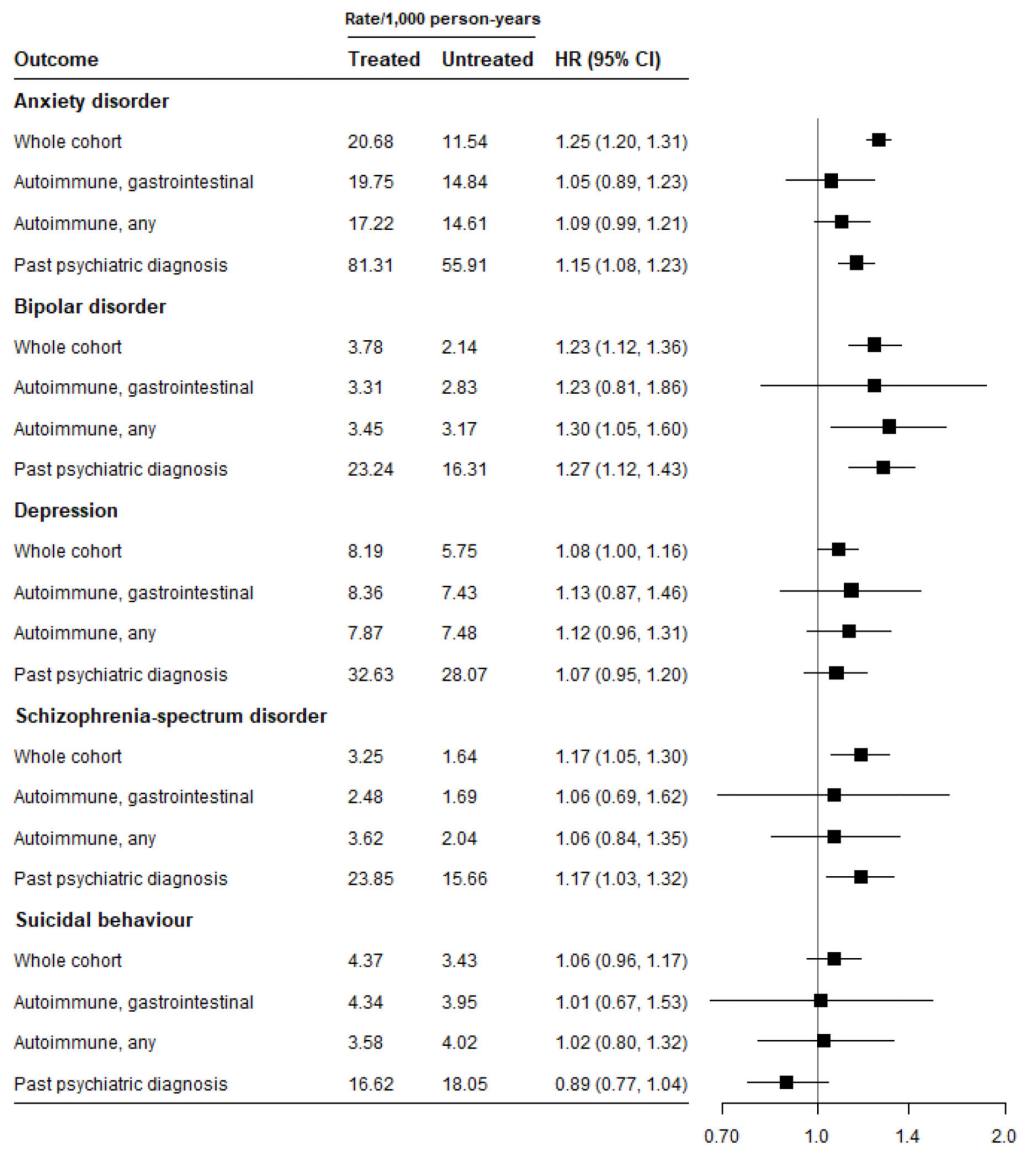
Psychiatric and suicidal behaviour outcomes in individuals dispensed a glucocorticoid (medication-only cohort) using a within-individual analysis, stratified by indication and past psychiatric diagnosis Rates are crude (unadjusted)

In the medication-only cohort, we found increased hazards for the main psychiatric disorders. Within-individual HRs in the overall cohort ranged between 1.06 and 1.25 (Figure 1), with no clear differences by age or sex strata (Tables S5 and S6). In addition, we found no strong evidence of an association with suicidal behaviour in within-individual analyses, overall (HR=1.06, 95% CI=0.96, 1.17; Figure 1), or when stratified by age or sex (Table S5 and S6). Absolute rates were highest in those with a psychiatric history for all outcomes (Figure 1). There was virtually no impact on results when excluding individuals ever dispensing an inhaled glucocorticoid during follow-up (Table S7).

We also carried out between-individual analyses in the medication-only cohort (Table S8). For all diagnostic outcomes, effect estimates were higher in between-individual compared to within-individual analyses. For example, the HR for anxiety disorders was 1.70 (95% CI=1.64, 1.77) and 1.25 (95% CI=1.20, 1.31) in between- and within-individual analyses, respectively.

To assess whether individuals with past mental health diagnosis were at greater risk of psychiatric outcomes, we considered a subset of the medication-only cohort where all individuals had received a diagnosis of one of the psychiatric outcomes considered in the analyses before start of follow-up (n=80,952, 7.3%; Table S8). Results were very similar to those in the main cohort (Figure 1).

We stratified the medication-only cohort by a diagnosis with an autoimmune disorder (N= 153,848; 13.9% of cohort) and a diagnosis with a gastrointestinal autoimmune disorder (N=58,318; 5.3% of cohort; Table S8). Associations were attenuated in both strata as compared to the overall cohort, with wide confidence intervals, apart from in the case of bipolar disorder outcomes in the autoimmune disorder subset. Point estimates were similar to those in the overall cohort for depression and bipolar disorder outcomes (Figure 1).

When considering risks in periods relative to treatment start, it appeared that the first 14 days since treatment start was associated with the highest hazard relative to untreated periods for anxiety (HR=1.37, 95% CI=1.27, 1.47) and bipolar disorders (HR=1.58, 95% CI=1.35, 1.85) (Table S9).

We further investigated the impact of average dose on the risk of the outcomes (Table S10). A high or medium dose was associated with higher risks of anxiety or bipolar disorder outcomes as compared to a low dose.

We also ran the main within-individual analyses in a cohort from the years 2006-2013 (N=680,215), where we could ensure that the dispensed medication was restricted to glucocorticoids (H02AB). There were no material differences in the results (Table S11).

Finally, we considered two cohorts defined by having an autoimmune disorder diagnosis (N=287,366) or a gastrointestinal autoimmune disorder diagnosis (N=96,029) rather than by medication receipt, in order to emulate two target trials assessing the effect on psychiatric and suicidal behaviour risks of initiating a glucocorticoid after an autoimmune diagnosis (Table S1). We applied IP weighting to balance baseline confounders between initiators and controls – an SMD of below 0.1 was achieved for all covariates after weighting (Table S2, Table S3), indicating sufficiently balanced covariates (27). In the intention-to-treat analyses, we found evidence of an increased risk of anxiety outcomes among initiators in the autoimmune disorder cohort, particularly early in follow-up (HR in the first 14 days of follow-up: 1.70, 95% CI=1.27, 1.28; Table 2). This was replicated when we stratified the cohort by past mental health disorder. We did not find evidence of other associations, and confidence intervals were wide. Anxiety outcomes had the highest cumulative incidence over follow-up. Those with past mental health disorders had substantially elevated cumulative risk of the outcomes – for example, 5.48% of initiators with a past mental health disorder had an anxiety outcome over the full 365 days of follow-up, compared to 1.15% in those without a history of a mental health disorder (Table 2). In per-protocol analyses, point estimates were similar but all confidence intervals included 1 (Table S12). We found no statistically significant associations for any of the outcomes in the gastrointestinal autoimmune disorder cohort in either the intention-to-treat or per-protocol analyses (Table S12; Table S13).

## Discussion

In this population-based nationwide study of 1,105,964 people, we found that glucocorticoid treatment was associated with modest elevated risks of unplanned specialist healthcare contacts due to anxiety, depressive, bipolar, or schizophrenia-spectrum disorders. The findings were consistent across men and women, different age bands, and individuals with a prior history of psychiatric disorder. Those with a history of psychiatric disorders had a higher absolute risk of all outcomes, and there may be a particularly elevated risk early in the treatment course. Glucocorticoid treatment was not associated with suicidal outcomes.

Our findings suggest that clinicians should be vigilant of the risk of acute psychiatric outcomes during glucocorticoid treatment, and aware that individuals with a history of psychiatric disorders have a particularly high baseline risk. Further research is necessary to predict who is at greatest risk of adverse psychiatric events during glucocorticoid treatments (1), and to investigate the impacts of dose and glucocorticoid subtype in more depth.

The lack of strong links between glucocorticoids and suicidal behaviour in our study is novel. A previous observational study using a between-individual design in UK primary care data found that glucocorticoid treatment was associated with a substantially increased hazard of suicidal behaviour (HR=6.9, 95% CI=4.5, 10.5) (3). This discrepancy with our results may be due to residual between-individual confounding in the previous paper. Our study also exclusively considered diagnoses from specialist care for the non-fatal component of suicidal behaviour, meaning our outcome likely represented more severe suicidal behaviours. Meanwhile, another investigation in Danish national health registers found a substantially elevated risk of suicide death after glucocorticoid treatment (4), though comparison with our results is difficult given that we considered a composite outcome including suicide attempts in addition to suicide death.

For the neuropsychiatric outcomes, our results are broadly consistent with prior evidence (28, 29). A recent systematic review and meta-analysis of evidence from observational studies and randomized controlled trials (RCTs) found elevated risk of depression and mania (30), though the included studies were highly heterogeneous. Compared to the previous large cohort study in UK primary care data discussed above, our associations were weaker – for example, we found a HR of 1.08 (95% CI=1.00, 1.16) for depression, compared to 1.8 (95% CI= 1.7, 1.9) in the prior study (3). The attenuated associations in our study may partly be due to our severe outcome definition. This is also likely to have reduced the prevalence of the outcomes. The proportion of glucocorticoid users with outcomes was lower in our study than in the prior review, where they found that 22% of all glucocorticoid users experienced events related to depressive disorder (30), as compared to 3.9% in our study.

Consistent with previous literature, we found that outcomes such as anxiety and bipolar disorder may have a higher risk of occurring in the first few weeks of treatment (31). Prior studies also find a dose-response relationship of glucocorticoids with the outcome (3–5). While our results may be consistent with such an effect, our method to estimate average dose made it difficult to separate the effects of dose from the impact of treatment time (see limitations section). Despite prior work finding that age is a moderator of the impact of glucocorticoid effects (3, 4, 32), our results did not support this.

In the between-individual analyses in the medication-only cohort, the associations with all outcomes were higher compared to the within-individual analyses, and there was a statistically significantly increased risk of suicidal behaviour. It is possible that individuals who have a higher baseline risk for psychiatric disorders also have a higher propensity for repeated treatment with glucocorticoids. This would inflate the associations of glucocorticoids with psychiatric outcomes when between-individual confounding is not fully accounted for.

We attempted to account for the impact of different indications for glucocorticoid treatment by stratifying on autoimmune and gastrointestinal autoimmune disorders in the medication-only cohort (Figure 1). Associations were attenuated in both diagnosis strata as compared to the overall cohort, with wide confidence intervals. The smaller number of individuals may make it hard to draw conclusions about outcomes that are already rare; alternatively, restricting to one type of indication may change the impact of different types of time-varying confounding in these cohorts.

We further triangulated our results by following individuals that were diagnosed with an autoimmune disorder or gastrointestinal autoimmune disorder and comparing psychiatric risks in those who did and did not initiate a glucocorticoid shortly after their diagnosis, using a target trial approach. Here, we found elevated risks of anxiety outcomes over the full follow-up in those with an autoimmune disorder diagnosis. Similarly to findings in the medication-only cohort, risks appeared particularly elevated over the first two weeks of treatment. No statistically significant associations were found for other outcomes or in the gastrointestinal autoimmune cohort, though these cohorts were limited in sample size and the confidence intervals were wide.

There is extensive evidence supporting a causal relationship between glucocorticoid treatment and psychiatric events (33). Dysregulation of the hypothalamic pituitary adrenal (HPA) axis, which controls glucocorticoid production in the body, has been linked to risk of anxiety and depression (34). Cortisol levels are elevated in individuals with first-episode psychosis (36), and higher cortisol levels have been associated with greater symptom severity in individuals with bipolar and schizophrenia disorders (37). Long-term exposure to excess glucocorticoids has also been linked to lower grey matter volume in the brain and higher rates of depression (38).

### Strengths and limitations

A key strength our use of a data linkage covering virtually the entire Swedish population. Further, to our knowledge, this is the first large-scale observational study to investigate psychiatric and suicidal behaviour risks of glucocorticoid treatment using both a self-controlled design and a target trial emulation approach.

Several limitations should be noted. First, while our within-individual analyses accounted for all time-invariant confounding, we could not account for unmeasured time-varying confounding, including time-varying confounding by indication. Glucocorticoids are prescribed for a wide range of disorders, including ones that are known to be associated with psychological distress. One important example is cancer, which is the indication for a significant proportion of individuals taking glucocorticoids (39). We did not have specific diagnostic information on cancer in our data linkage. If prescribed a glucocorticoid due to e.g. cancer relapse, any apparent psychiatric risk of treatment may be due to the distress of dealing with a serious somatic disorder. This is also likely to be true of autoimmune disorders, which are associated with discomfort and stress – there are bidirectional relationships between autoimmune and psychiatric disorders (40). However, it is also possible that certain chronic symptoms of these disorders are alleviated during glucocorticoid treatment. Second, we did not have information on diagnoses given in primary care, and hence could not estimate risks of less serious psychiatric and behavioural outcomes. However, our inclusion of unplanned specialist care diagnoses meant that we have investigated more severe events that are of greatest clinical interest. Many prior studies consider less severe definitions of the outcomes, making our study a useful addition to the literature. Third, our treatment period definition may misclassify follow-up time as either treated or untreated: we do not know whether individuals consumed their medication after purchase. This means our analyses estimate a modified intention-to-treat effect, which is expected to bias results toward the null (41). The classification of any given time period as treated or untreated also relied on whether a medication was dispensed at a future point in time – i.e. within 120 days since the last dispensed prescription. This may induce bias if, for example, the occurrence of an outcome of interest influences future prescribing behaviour. However, the intention-to-treat analyses in the target trial emulations were not subject to this bias. We also did not have structured information on the prescribed daily dose, but calculated average daily dose by taking cumulative dispensed DDDs divided by treatment time. This was subject to the same issues of relying on future prescription information as described above, and made it difficult to separate effects of treatment duration from those of treatment dose. Finally, while our results derive from a large and nationally representative cohort with comprehensive information on psychiatric diagnoses received in specialist care, they may not be generalizable to other time periods, national settings, or clinical contexts.

### Conclusions

In conclusion, we found that glucocorticoid treatment was associated with an elevated risks for psychiatric events leading to unplanned specialist psychiatric care, but not suicidal behaviour. Risks may be particularly elevated during the first few weeks of treatment, though we could not fully distinguish between time and dosage effects. Absolute rates were highest among those with a past psychiatric history, suggesting this population might require more clinical attention. Clinicians should be vigilant of psychiatric outcomes during glucocorticoid treatment, which include the onset and relapse of common psychiatric disorders.

## Supplementary Material

Supplementary tables and figures

## Figures and Tables

**Table 1:** Characteristics of the cohort of individuals dispensed a glucocorticoid (medication-only cohort)

	Overall (N = 1,105,964)	Female (N = 642,567)	Male (N = 463,397)
**Baseline age^a^ (%)**	**N (%)**	**N (%)**	**N (%)**
**15-24 years**	391888 (35.4%)	224461 (34.9%)	167427 (36.1%)
**25-34 years**	263173 (23.8%)	152607 (23.7%)	110566 (23.9%)
**35-44 years**	304778 (27.6%)	177141 (27.6%)	127637 (27.5%)
**45-54 years**	146125 (13.2%)	88358 (13.8%)	57767 (12.5%)
**Mean length of full follow-up, yrs**	12.0	12.0	12.0
**No. individuals with event (%)**			
Anxiety disorders	76826 (6.9%)	52181 (8.1%)	24645 (5.3%)
Bipolar disorder	8805 (0.8%)	6442 (1%)	2363 (0.5%)
Depressive disorders	43410 (3.9%)	29614 (4.6%)	13796 (3%)
Schizophrenia-spectrum disorders	6581 (0.6%)	3465 (0.5%)	3116 (0.7%)
Suicidal behaviour	29063 (2.6%)	17725 (2.8%)	11338 (2.4%)

a At start of follow-up

**Table 2:** Intention-to-treat analysis of psychiatric and suicidal behaviour outcomes in individual with an autoimmune disorder diagnosis initiating a glucocorticoid versus those who do not (indication cohort), stratified by past psychiatric diagnosis

Outcome	Time	Overall cohort (N = 287,366)			Past mental health cohort (N= 39,742)		
		Cumulative risk (%)		Hazard Ratio	Cumulative Risk (%)		Hazard Ratio
		Initiators	Controls		Initiators	Controls	
**Anxiety**	**14 days**	0.10 (0.07,0.13)	0.06 (0.06,0.07)	1.70 (1.27,2.27)	0.59 (0.40,0.79)	0.31 (0.28,0.35)	1.90 (1.35,2.66)
	**120 days**	0.57 (0.48,0.66)	0.40 (0.38,0.42)	1.55 (1.25,1.93)	3.12 (2.49,3.75)	1.96 (1.81,2.11)	1.73 (1.33,2.24)
	**365 days**	1.15 (1.01,1.28)	0.99 (0.95,1.02)	1.34 (1.16,1.55)	5.48 (4.60,6.37)	4.51 (4.29,4.73)	1.47 (1.22,1.77)
**Bipolar** **disorder**	**14 days**	0.02 (0.01,0.03)	0.01 (0.01,0.02)	1.22 (0.62,2.40)	0.11 (0.03,0.19)	0.10 (0.08,0.12)	1.11 (0.53,2.31)
	**120 days**	0.10 (0.06,0.14)	0.09 (0.08,0.10)	1.16 (0.67,2.00)	0.60 (0.31,0.90)	0.60 (0.52,0.68)	1.05 (0.58,1.89)
	**365 days**	0.20 (0.14,0.26)	0.18 (0.17,0.20)	1.10 (0.75,1.62)	1.28 (0.86,1.70)	1.25 (1.13,1.38)	1.00 (0.66,1.51)
**Depression**	**14 days**	0.03 (0.02,0.05)	0.04 (0.03,0.04)	0.92 (0.57,1.46)	0.18 (0.08,0.27)	0.20 (0.18,0.23)	0.86 (0.48,1.56)
	**120 days**	0.24 (0.18,0.31)	0.24 (0.22,0.26)	0.97 (0.68,1.39)	1.22 (0.84,1.60)	1.28 (1.17,1.40)	0.91 (0.58,1.41)
	**365 days**	0.74 (0.63,0.85)	0.61 (0.58,0.64)	1.08 (0.88,1.34)	3.28 (2.63,3.92)	2.96 (2.79,3.14)	1.00 (0.76,1.31)
**Schizophrenia** **spectrum** **disorders**	**14 days**	0.02 (0.00,0.03)	0.02 (0.01,0.02)	1.10 (0.52,2.31)	0.11 (0.03,0.20)	0.11 (0.09,0.13)	1.03 (0.44,2.44)
	**120 days**	0.10 (0.06,0.15)	0.09 (0.08,0.10)	1.14 (0.66,1.96)	0.66 (0.34,0.98)	0.60 (0.52,0.67)	1.08 (0.56,2.05)
	**365 days**	0.18 (0.13,0.24)	0.19 (0.17,0.20)	1.11 (0.77,1.60)	1.21 (0.79,1.62)	1.20 (1.09,1.31)	1.08 (0.70,1.67)
**Suicidal** **behaviour**	**14 days**	0.01 (0.00,0.02)	0.02 (0.01,0.02)	0.80 (0.39,1.64)	0.09 (0.02,0.15)	0.09 (0.07,0.10)	1.02 (0.48,2.16)
	**120 days**	0.10 (0.06,0.13)	0.12 (0.11,0.13)	0.79 (0.46,1.38)	0.53 (0.29,0.78)	0.60 (0.53,0.68)	0.94 (0.52,1.71)
	**365 days**	0.32 (0.25,0.40)	0.33 (0.31,0.36)	0.84 (0.61,1.17)	1.42 (0.99,1.85)	1.55 (1.43,1.68)	0.88 (0.59,1.32)

## Data Availability

The Public Access to Information and Secrecy Act in Sweden prohibits us from making individual-level data publicly available. Researchers may apply for individual-level data from the National Board of Health and Welfare (registerservice@socialstyrelsen.se) for data from the Patient Register, the Prescribed Drug Register and the Cause of Death Register; and to Statistics Sweden (mikrodata@scb.se) for data from the Total Population Register.

## Relevance Statement

In a cohort study of 1,105,964 individuals ages 15-54 years prescribed and dispensed glucocorticoids in Sweden 2006-2020, we found statistically significantly elevated risks for incident diagnoses and relapses of anxiety, bipolar disorder, depression, and schizophrenia spectrum disorders (hazard ratios ranging from 1.08 to 1.25) during glucocorticoid treatment, but no increased risk for suicidal outcomes. Absolute rates were higher in individuals with a history of psychiatric disorders. Clinical awareness of psychiatric risks during glucocorticoid treatment is warranted, particularly in individuals with a psychiatric history.
