# Supplementary material for: Oral glucocorticoids and the risk of psychiatric and suicidal behaviour outcomes: a population-based cohort study
Source: Br J Psychiatry. Author manuscript; Available in PMC 2026 Jul 1. (PMC7617852; doi:10.1192/bjp.2025.128)
Supplement: Supplementary tables and figures [file EMS204641-supplement-Supplementary_tables_and_figures.docx]

Supplement: Oral glucocorticoids and risk of psychiatric and suicidal behaviour outcomes: a population-based cohort study

**Contents**

[Table S1. Target trial emulation, indication cohorts 2](#_Toc198308259)

[Table S2. ICD-10 codes for autoimmune disorders 4](#_Toc198308260)

[Table S3. Characteristics of the individuals followed since an autoimmune disorder diagnosis (indication cohort) 5](#_Toc198308261)

[Table S4. Characteristics of the individuals followed since a gastrointestinal autoimmune disorder diagnosis (indication cohort) 6](#_Toc198308262)

[Table S5. Psychiatric and suicidal behaviour outcomes in individuals dispensed a glucocorticoid (medication-only cohort) using a within-individual analysis, stratified by sex 7](#_Toc198308263)

[Table S6. Psychiatric and suicidal behaviour outcomes in individuals dispensed a glucocorticoid (medication-only cohort) using a within-individual analysis, stratified by age category 8](#_Toc198308264)

[Table S7. Psychiatric and suicidal behaviour outcomes in individuals dispensed a glucocorticoid (medication-only cohort) using a within-individual analysis, excluding individuals ever dispensing an inhaled glucocorticoid during follow-up 9](#_Toc198308265)

[Table S8. Psychiatric and suicidal behaviour outcomes in individuals dispensed a glucocorticoid (medication-only cohort) using a within-individual or between-individual analysis, stratified by indication and past psychiatric diagnosis 10](#_Toc198308266)

[Table S9. Psychiatric and suicidal behaviour outcomes in individuals dispensed a glucocorticoid (medication-only cohort) using a within-individual analysis, by treatment periods 11](#_Toc198308267)

[Table S10. Associations of glucocorticoid treatment with the risk of psychiatric and suicidal behaviour outcomes at low, medium, and high average dosage 12](#_Toc198308268)

[Table S11. Psychiatric and suicidal behaviour outcomes in individuals dispensed a glucocorticoid (medication-only cohort) using a within-individual analysis in the data linkage covering 2006-2020, compared to the data linkage covering 2006-2013 only 13](#_Toc198308269)

[Table S12. Per-protocol analyses in individuals with an autoimmune disorder or gastrointestinal autoimmune disorder diagnosis (indication cohorts) 14](#_Toc198308270)

[Table S13. Intention-to-treat analyses in individuals with a gastrointestinal autoimmune disorder diagnosis (indication cohort) 15](#_Toc198308271)

## Table S1. Target trial emulation, indication cohorts

| Component | Target trial specifications | Target trial emulation |
| --- | --- | --- |
| **Eligibility criteria** | - Age 15-54 years old 1 January 2006 to November 30 2019. - Any autoimmune disorder cohort: first diagnosis of any autoimmune disorder 1 January 2006 to 30 November 2019. - Gastrointestinal autoimmune disorder cohort: first diagnosis of a gastrointestinal autoimmune disorder 1 January 2006 to 30 November 2019. - No glucocorticoid (H02) prescriptions within 180 days before the autoimmune diagnosis. | Same as target trial. |
| **Treatment strategies** | 1. Initiation of a glucocorticoid (H02) within 28 days of the autoimmune diagnosis then continuing to take the treatment for 52 weeks. 2. No initiation of a glucocorticoid within 28 days of the autoimmune diagnosis and then remaining off treatment for 52 weeks. | Same as target trial.    Follow-up starts at the time an individual collects a prescription in individuals assigned as initiators at baseline. The number of days between the autoimmune diagnosis and the dispensation of a glucocorticoid prescription in initiators is randomly assigned to controls – the start of follow-up in controls occurs on this number of days after their autoimmune diagnosis. |
| **Treatment assignment** | At baseline, individuals are assigned to a strategy at random and are aware of their assigned strategy. | We classify individuals according to the strategy they are compatible with at baseline.  Assignment of treatment strategy is assumed to be random within levels of:  sex, age category, year of follow-up start, highest level of attained education of individual and their parents, highest income category between individual and parents, and diagnoses (anxiety, bipolar disorder, depression, schizophrenia spectrum disorders, substance use disorder, and a history of self-harm). |
| **Outcomes** | Unplanned specialist inpatient or specialist outpatient contacts for:  Anxiety disorder  ICD-10: F4  Bipolar disorder  ICD-10: F25.0, F30, F31, F340  Depression  ICD-10: F32, F33, F34 excl. F34.0, F38, F39  Schizophrenia spectrum disorders  ICD-10: F2, excl. F25.0  Suicidal behaviour  ICD-10: X60 - X84; Y10 - Y34 | Same as for target trial. |
| **Follow-up** | Follow-up starts at randomization and ends at (whichever occurs first):   - 52 weeks after randomization - Outcome - Death - Emigration | Same as for target trial. |
| **Causal contrasts** | 1. Intention-to-treat effect at 14, 120, and 365 days of follow-up. 2. Per-protocol effect at 14, 120, and 365 days of follow-up. | Observational analog of intention-to-treat and per-protocol effect. |
| **Analysis plan** | Separate analyses are run for each outcome type.  **ITT:** Individuals are assumed to remain on the treatment they are assigned at baseline throughout follow-up.  **Per-protocol:** Individuals are censored when they cease adhering to the treatment they are assigned at baseline. To mitigate bias from this censoring, we apply IPW weighting for adherence to treatment using baseline confounders and any specialist outpatient or inpatient healthcare contact for any of the following over follow-up: alcohol use disorder, substance use disorder, depression, anxiety disorders, bipolar disorders, schizophrenia spectrum disorders, or self-harm (see outcome ICD-10 codes). Analyses of a specific psychiatric outcome do not consider healthcare contacts for that outcome type over follow-up as a confounder.  We run subgroup analyses by baseline history of any of the events considered as outcomes (past psychiatric diagnosis) for the intention-to-treat analyses. | The same analyses with sequential emulation and additional adjustment for baseline covariates.  Same subgroup analyses. |

## Table S2. ICD-10 codes for autoimmune disorders

|  | **ICD-10** | **Name** |
| --- | --- | --- |
|  | D68.61 | Antiphospholipid syndrome |
|  | D69.0 | Allergic purpura |
|  | D69.3 | Immune thrombocytopenic purpura |
|  | E05.0 | Thyrotoxicosis with diffuse goiter |
|  | E06.3 | Autoimmune thyroiditis |
|  | E31.0 | Autoimmune polyglandular failure |
|  | G04 | Encephalitis, myelitis and encephalomyelitis |
|  | G13.1 | Other systemic atrophy primarily affecting central nervous system in neoplastic disease |
|  | G35 | Multiple sclerosis |
|  | G61.0 | Guillain-Barre syndrome |
|  | G70.0 | Myasthenia gravis |
|  | I00-I02 | Acute rheumatic fever |
|  | A38.9 | Scarlet fever |
|  | K50-K51 | Crohn's disease; Ulcerative colitis |
|  | K74.3 | Primary biliary cirrhosis |
|  | K90.0 | Celiac disease |
|  | L10.0 | Pemphigus vulgaris |
|  | L12.0 | Bullous pemphigoid |
|  | L13 | Dermatitis herpetiformis |
|  | L40 | Psoriasis |
|  | L63 | Alopecia areata |
|  | M06 | Rheumatoid arthritis |
|  | M30.0-M30.1 | Polyarteritis nodosa; Polyarteritis nodosa with lung involvement |
|  | M30.3 | Mucocutaneous lymph node syndrome |
|  | M31.1 | Thrombotic microangiopathy |
|  | M31.5 | Giant cell arteritis with polymyalgia rheumatica |
|  | M31.7 | Microscopic polyangiitis |
|  | M32 | Systemic lupus erythematosus |
|  | M33.9 | Dermatopolymyositis |
|  | M34 | Systemic sclerosis |
|  | M35.0 | Sjogren syndrome |
|  | M35.1 | Other overlap syndromes |
|  | M35.2-M35.3 | Behcet's disease; Polymyalgia rheumatica |
|  | N00-N01 | Acute nephritic syndrome; Rapidly progressive nephritic syndrome |
|  | N03 | Chronic nephritic syndrome |
|  | N05 | Unspecified nephritic syndrome |
| **Gastrointestinal autoimmune disorders** | K50 | Crohn’s disease |
|  | K51 | Ulcerative colitis |
|  | K743 | Primary biliary cirrhosis |
|  | K900 | Coeliac disease |
|  | L13 | Dermatitis herpetiformis |

## Table S3. Characteristics of the individuals followed since an autoimmune disorder diagnosis (indication cohort)

|  | **Before IP weighting** | | | **After IP weighting** | | |
| --- | --- | --- | --- | --- | --- | --- |
|  | **Initiator** | **Non-initiator** | **SMD^a^** | **Initiator** | **Non-initiator** | **SMD^a^** |
| **N** | 22621 | 264745 |  | 22122.4 | 264743.3 |  |
| **Sex (%)** | 12132 (53.6) | 159862 (60.4) | 0.137 | 13051.7 (59.0) | 158462.3 (59.9) | 0.017 |
| **Age category (years) (%)** |  |  | 0.100 |  |  | 0.016 |
| 15-24 | 5735 (25.4) | 58872 (22.2) |  | 5080.1 (23.0) | 59515.8 (22.5) |  |
| 25-34 | 5586 (24.7) | 61036 (23.1) |  | 5192.4 (23.5) | 61377.2 (23.2) |  |
| 35-44 | 5348 (23.6) | 70637 (26.7) |  | 5742.4 (26.0) | 70006.8 (26.4) |  |
| 45-54 | 5952 (26.3) | 74200 (28.0) |  | 6107.4 (27.6) | 73843.4 (27.9) |  |
| **Year of follow-up start (%)** |  |  | 0.161 |  |  | 0.057 |
| 2006 | 2938 (13.0) | 49008 (18.5) |  | 3526.4 (15.9) | 47857.3 (18.1) |  |
| 2007 | 1925 (8.5) | 23579 (8.9) |  | 1998.3 (9.0) | 23497.2 (8.9) |  |
| 2008 | 1687 (7.5) | 19847 (7.5) |  | 1692.8 (7.7) | 19839.4 (7.5) |  |
| 2009 | 1554 (6.9) | 17283 (6.5) |  | 1485.0 (6.7) | 17353.8 (6.6) |  |
| 2010 | 1515 (6.7) | 16031 (6.1) |  | 1388.7 (6.3) | 16164.4 (6.1) |  |
| 2011 | 1525 (6.7) | 16171 (6.1) |  | 1405.4 (6.4) | 16303.1 (6.2) |  |
| 2012 | 1540 (6.8) | 17015 (6.4) |  | 1467.3 (6.6) | 17094.8 (6.5) |  |
| 2013 | 1466 (6.5) | 16412 (6.2) |  | 1401.4 (6.3) | 16469.7 (6.2) |  |
| 2014 | 1460 (6.5) | 15840 (6.0) |  | 1365.3 (6.2) | 15937.6 (6.0) |  |
| 2015 | 1358 (6.0) | 15363 (5.8) |  | 1309.9 (5.9) | 15404.3 (5.8) |  |
| 2016 | 1500 (6.6) | 15236 (5.8) |  | 1342.3 (6.1) | 15419.2 (5.8) |  |
| 2017 | 1423 (6.3) | 14997 (5.7) |  | 1294.9 (5.9) | 15126.2 (5.7) |  |
| 2018 | 1396 (6.2) | 14402 (5.4) |  | 1256.3 (5.7) | 14554.1 (5.5) |  |
| 2019 | 1334 (5.9) | 13561 (5.1) |  | 1188.3 (5.4) | 13722.0 (5.2) |  |
| **Family income category (%)** |  |  | 0.027 |  |  | 0.010 |
| <0 | 210 (0.9) | 2396 (0.9) |  | 209.0 (0.9) | 2401.5 (0.9) |  |
| 0 | 144 (0.6) | 1368 (0.5) |  | 120.9 (0.5) | 1392.8 (0.5) |  |
| 0-p20 | 2107 (9.3) | 23416 (8.8) |  | 2011.3 (9.1) | 23516.6 (8.9) |  |
| p20-p80 | 13200 (58.4) | 153796 (58.1) |  | 12871.0 (58.2) | 153849.6 (58.1) |  |
| >p80 | 6960 (30.8) | 83769 (31.6) |  | 6910.1 (31.2) | 83582.9 (31.6) |  |
| **Education category (%)** |  |  | 0.036 |  |  | 0.011 |
| Primary | 1647 (7.3) | 18543 (7.0) |  | 1585.5 (7.2) | 18603.2 (7.0) |  |
| Secondary | 10204 (45.1) | 116356 (44.0) |  | 9781.9 (44.2) | 116594.6 (44.0) |  |
| Post-secondary | 10294 (45.5) | 124915 (47.2) |  | 10318.3 (46.6) | 124563.2 (47.1) |  |
| NA | 476 (2.1) | 4931 (1.9) |  | 436.6 (2.0) | 4982.3 (1.9) |  |
| **Depression (%)** | 1398 (6.2) | 17011 (6.4) | 0.010 | 1418.3 (6.4) | 16961.0 (6.4) | <0.001 |
| **Bipolar disorder diagnosis (%)** | 186 (0.8) | 2778 (1.0) | 0.024 | 210.6 (1.0) | 2730.6 (1.0) | 0.008 |
| **Anxiety disorder diagnosis (%)** | 1888 (8.3) | 23454 (8.9) | 0.018 | 1945.7 (8.8) | 23348.2 (8.8) | 0.001 |
| **Schizophrenia diagnosis (%)** | 169 (0.7) | 2848 (1.1) | 0.035 | 197.9 (0.9) | 2779.3 (1.0) | 0.016 |
| **Substance abuse disorder (excl. alcohol) diagnosis (%)** | 595 (2.6) | 6743 (2.5) | 0.005 | 570.4 (2.6) | 6760.6 (2.6) | 0.002 |
| **Suicide history (%)** | 770 (3.4) | 8976 (3.4) | 0.001 | 751.0 (3.4) | 8978.6 (3.4) | <0.001 |
| ^a^ SMD: Standardized Mean Difference | | | | | | |

## Table S4. Characteristics of the individuals followed since a gastrointestinal autoimmune disorder diagnosis (indication cohort)

|  | **Before IP weighting** | | | **After IP weighting** | | |
| --- | --- | --- | --- | --- | --- | --- |
|  | **Initiator** | **Non-initiator** | **SMD^a^** | **Initiator** | **Non-initiator** | **SMD^a^** |
| **N** | 12360 | 83669 |  | 12155.4 | 83663.2 |  |
| **Sex (%)** | 5400 (43.7) | 47526 (56.8) | 0.265 | 6639.9 (54.6) | 46122.8 (55.1) | 0.010 |
| **Age category (years) (%)** |  |  | 0.135 |  |  | 0.012 |
| 15-24 | 4148 (33.6) | 28786 (34.4) |  | 4143.3 (34.1) | 28690.4 (34.3) |  |
| 25-34 | 3464 (28.0) | 18856 (22.5) |  | 2880.5 (23.7) | 19439.3 (23.2) |  |
| 35-44 | 2559 (20.7) | 18638 (22.3) |  | 2690.0 (22.1) | 18472.8 (22.1) |  |
| 45-54 | 2189 (17.7) | 17389 (20.8) |  | 2441.6 (20.1) | 17060.7 (20.4) |  |
| **Year of follow-up start (%)** |  |  | 0.219 |  |  | 0.034 |
| 2006 | 1782 (14.4) | 18380 (22.0) |  | 2390.4 (19.7) | 17570.9 (21.0) |  |
| 2007 | 1130 (9.1) | 8832 (10.6) |  | 1296.5 (10.7) | 8682.7 (10.4) |  |
| 2008 | 981 (7.9) | 6683 (8.0) |  | 993.0 (8.2) | 6678.5 (8.0) |  |
| 2009 | 853 (6.9) | 5367 (6.4) |  | 798.7 (6.6) | 5417.6 (6.5) |  |
| 2010 | 804 (6.5) | 4714 (5.6) |  | 709.3 (5.8) | 4807.2 (5.7) |  |
| 2011 | 800 (6.5) | 4820 (5.8) |  | 722.7 (5.9) | 4895.8 (5.9) |  |
| 2012 | 758 (6.1) | 4474 (5.3) |  | 675.8 (5.6) | 4558.7 (5.4) |  |
| 2013 | 790 (6.4) | 4415 (5.3) |  | 659.8 (5.4) | 4531.3 (5.4) |  |
| 2014 | 806 (6.5) | 4379 (5.2) |  | 676.5 (5.6) | 4518.0 (5.4) |  |
| 2015 | 673 (5.4) | 4230 (5.1) |  | 627.5 (5.2) | 4271.1 (5.1) |  |
| 2016 | 768 (6.2) | 4377 (5.2) |  | 666.6 (5.5) | 4483.0 (5.4) |  |
| 2017 | 737 (6.0) | 4313 (5.2) |  | 637.6 (5.2) | 4396.7 (5.3) |  |
| 2018 | 759 (6.1) | 4235 (5.1) |  | 634.1 (5.2) | 4347.9 (5.2) |  |
| 2019 | 719 (5.8) | 4450 (5.3) |  | 666.8 (5.5) | 4503.9 (5.4) |  |
| **Family income category (%)** |  |  | 0.061 |  |  | 0.014 |
| <0 | 88 (0.7) | 550 (0.7) |  | 88.0 (0.7) | 557.2 (0.7) |  |
| 0 | 64 (0.5) | 314 (0.4) |  | 48.2 (0.4) | 329.0 (0.4) |  |
| 0-p20 | 1052 (8.5) | 5860 (7.0) |  | 910.6 (7.5) | 6026.2 (7.2) |  |
| p20-p80 | 7295 (59.0) | 50299 (60.1) |  | 7288.0 (60.0) | 50179.4 (60.0) |  |
| >p80 | 3861 (31.2) | 26646 (31.8) |  | 3820.5 (31.4) | 26571.4 (31.8) |  |
| **Education category (%)** |  |  | 0.067 |  |  | 0.013 |
| Primary | 765 (6.2) | 4589 (5.5) |  | 699.3 (5.8) | 4668.5 (5.6) |  |
| Secondary | 5612 (45.4) | 36489 (43.6) |  | 5345.2 (44.0) | 36676.6 (43.8) |  |
| Post-secondary | 5775 (46.7) | 41530 (49.6) |  | 5937.1 (48.8) | 41210.6 (49.3) |  |
| NA | 208 (1.7) | 1061 (1.3) |  | 173.8 (1.4) | 1107.5 (1.3) |  |
| **Depression (%)** | 711 (5.8) | 4688 (5.6) | 0.006 | 700.8 (5.8) | 4706.3 (5.6) | 0.006 |
| **Bipolar disorder diagnosis (%)** | 79 (0.6) | 596 (0.7) | 0.009 | 86.4 (0.7) | 588.2 (0.7) | 0.001 |
| **Anxiety disorder diagnosis (%)** | 950 (7.7) | 6472 (7.7) | 0.002 | 960.2 (7.9) | 6469.3 (7.7) | 0.006 |
| **Schizophrenia diagnosis (%)** | 81 (0.7) | 679 (0.8) | 0.018 | 88.9 (0.7) | 661.9 (0.8) | 0.007 |
| **Substance abuse disorder (excl. alcohol) diagnosis (%)** | 298 (2.4) | 1625 (1.9) | 0.032 | 254.7 (2.1) | 1676.4 (2.0) | 0.006 |
| **Suicide history (%)** | 406 (3.3) | 2495 (3.0) | 0.017 | 372.5 (3.1) | 2527.3 (3.0) | 0.003 |
| ^a^ SMD: Standardized Mean Difference | | | | | | |

## Table S5. Psychiatric and suicidal behaviour outcomes in individuals dispensed a glucocorticoid (medication-only cohort) using a within-individual analysis, stratified by sex

|  | **Males (N=463,397)** | | **Females (N=642,567)** | |
| --- | --- | --- | --- | --- |
| **Outcome** | **N events** | **HR (95% CI)** | **N events** | **HR (95% CI)** |
| **Anxiety** | 48,045 | 1.26 (1.15, 1.38) | 107,863 | 1.25 (1.19, 1.32) |
| **Bipolar disorder** | 7,742 | 1.17 (0.94, 1.45) | 21,156 | 1.25 (1.12, 1.40) |
| **Depression** | 23,843 | 1.00 (0.86, 1.15) | 53,217 | 1.12 (1.02, 1.22) |
| **Psychotic disorder** | 9,996 | 1.19 (0.99, 1.42) | 12,234 | 1.16 (1.02, 1.33) |
| **Suicidal behaviour** | 15,773 | 0.97 (0.80, 1.18) | 30,111 | 1.09 (0.97, 1.23) |

## Table S6. Psychiatric and suicidal behaviour outcomes in individuals dispensed a glucocorticoid (medication-only cohort) using a within-individual analysis, stratified by age category

|  | **Age category^a^** | | | | | | | |
| --- | --- | --- | --- | --- | --- | --- | --- | --- |
|  | **15-24 years**  **(N=391,888)** | | **25-34 years**  **(N=569,909)** | | **35-44 years**  **(N=658,122)** | | **45-54 years**  **(N=569,912)** | |
| **Outcome** | **N events** | **HR (95% CI)** | **N events** | **HR (95% CI)** | **N events** | **HR (95% CI)** | **N events** | **HR (95% CI)** |
| **Anxiety** | 34231 | 1.23 (1.09, 1.39) | 44293 | 1.22 (1.09, 1.35) | 41706 | 1.38 (1.25, 1.53) | 35678 | 1.24 (1.13, 1.37) |
| **Bipolar disorder** | 3897 | 1.04 (0.77, 1.41) | 8086 | 1.41 (1.13, 1.77) | 8518 | 1.03 (0.81, 1.32) | 8397 | 1.41 (1.19, 1.67) |
| **Depression** | 16261 | 1.05 (0.85, 1.30) | 20370 | 1.26 (1.03, 1.53) | 21124 | 1.05 (0.89, 1.24) | 19305 | 1.07 (0.92, 1.24) |
| **Psychotic disorder** | 2102 | 1.03 (0.59, 1.79) | 5155 | 1.03 (0.74, 1.42) | 6948 | 1.18 (0.94, 1.47) | 8025 | 1.16 (0.98, 1.38) |
| **Suicidal behaviour** | 14456 | 1.19 (0.96, 1.46) | 11458 | 1.20 (0.94, 1.54) | 10438 | 0.91 (0.71, 1.16) | 9532 | 0.93 (0.72, 1.19) |

^a^ Individuals may contribute to more than one age category over follow-up

## Table S7. Psychiatric and suicidal behaviour outcomes in individuals dispensed a glucocorticoid (medication-only cohort) using a within-individual analysis, excluding individuals ever dispensing an inhaled glucocorticoid during follow-up

| **Outcome** | **Overall cohort** | **Excluding individuals dispensing an inhaled glucocorticoid^a^** |
| --- | --- | --- |
|  | **HR (95% CI)** | **HR (95% CI)** |
| **Anxiety** | 1.25 (1.20, 1.31) | 1.29 (1.21, 1.37) |
| **Bipolar disorder** | 1.23 (1.12, 1.36) | 1.20 (1.04, 1.38) |
| **Depression** | 1.08 (1.00, 1.16) | 1.11 (1.01, 1.22) |
| **Psychotic disorder** | 1.17 (1.05, 1.30) | 1.14 (0.99, 1.32) |
| **Suicidal behaviour** | 1.06 (0.96, 1.17) | 1.12 (0.97, 1.29) |
| ^a^ Inhaled glucocorticoids ATC code: R03BA | | |

## Table S8. Psychiatric and suicidal behaviour outcomes in individuals dispensed a glucocorticoid (medication-only cohort) using a within-individual or between-individual analysis, stratified by indication and past psychiatric diagnosis

|  | **Overall cohort**  **(N=1,105,964)** | | **Past psychiatric diagnosis**  **(N=80,952)** | | **Autoimmune disorder cohort**  **(N= 153,848)** | | **Gastrointestinal autoimmune disorder cohort**  **(N= 58,318)** | |
| --- | --- | --- | --- | --- | --- | --- | --- | --- |
|  | **Between-individual^a^**  **HR (95% CI)** | **Within-individual^b^**  **HR (95% CI)** | **Between-individual^a^**  **HR (95% CI)** | **Within-individual^b^**  **HR (95% CI)** | **Between-individual^a^**  **HR (95% CI)** | **Within-individual^b^**  **HR (95% CI)** | **Between-individual^a^**  **HR (95% CI)** | **Within-individual^b^**  **HR (95% CI)** |
| **Anxiety** | 1.70 (1.64, 1.77) | 1.25 (1.20, 1.31) | 1.36 (1.28, 1.45) | 1.15 (1.08, 1.23) | 1.03 (0.95, 1.11) | 1.09 (0.99, 1.21) | 1.12 (0.99, 1.27) | 1.05 (0.89, 1.23) |
| **Bipolar disorder** | 1.49 (1.34, 1.66) | 1.23 (1.12, 1.36) | 1.20 (1.04, 1.38) | 1.27 (1.12, 1.43) | 0.80 (0.65, 0.98) | 1.30 (1.05, 1.60) | 0.86 (0.59, 1.26) | 1.23 (0.81, 1.86) |
| **Depression** | 1.32 (1.25, 1.39) | 1.08 (1.00, 1.16) | 1.08 (0.99, 1.17) | 1.07 (0.95, 1.20) | 0.88 (0.79, 0.98) | 1.12 (0.96, 1.31) | 0.94 (0.79, 1.11) | 1.13 (0.87, 1.46) |
| **Schizophrenia spectrum disorder** | 1.51 (1.32, 1.71) | 1.17 (1.05, 1.30) | 1.11 (0.97, 1.27) | 1.17 (1.03, 1.32) | 1.19 (0.92, 1.54) | 1.06 (0.84, 1.35) | 1.07 (0.72, 1.58) | 1.06 (0.69, 1.62) |
| **Suicidal behaviour** | 1.21 (1.13, 1.30) | 1.06 (0.96, 1.17) | 0.92 (0.82, 1.03) | 0.89 (0.77, 1.04) | 0.77 (0.67, 0.89) | 1.02 (0.80, 1.32) | 0.90 (0.70, 1.15) | 1.01 (0.67, 1.53) |

^a^ Adjusted for age and sex

^b^ Adjusted for age

## Table S9. Psychiatric and suicidal behaviour outcomes in individuals dispensed a glucocorticoid (medication-only cohort) using a within-individual analysis, by treatment periods

|  |  | **Time since treatment start** | | | |
| --- | --- | --- | --- | --- | --- |
|  |  | **<14 days** | **14-119 days** | **120-364 days** | **>364 days** |
|  |  | **HR (95% CI)** | **HR (95% CI)** | **HR (95% CI)** | **HR (95% CI)** |
| **Anxiety** | | 1.37 (1.27, 1.47) | 1.23 (1.15, 1.31) | 1.14 (1.04, 1.25) | 1.19 (1.05, 1.35) |
| **Bipolar disorder** | | 1.58 (1.35, 1.85) | 1.06 (0.91, 1.24) | 1.00 (0.82, 1.23) | 1.32 (1.02, 1.70) |
| **Depression** | | 1.17 (1.03, 1.32) | 0.98 (0.87, 1.09) | 1.20 (1.04, 1.38) | 1.03 (0.85, 1.25) |
| **Schizophrenia spectrum disorder** | | 1.18 (0.97, 1.42) | 1.11 (0.94, 1.31) | 1.25 (1.02, 1.54) | 1.19 (0.94, 1.51) |
| **Suicidal behaviour** | | 1.07 (0.90, 1.27) | 1.02 (0.88, 1.19) | 1.14 (0.93, 1.40) | 0.98 (0.72, 1.34) |

## Table S10. Associations of glucocorticoid treatment with the risk of psychiatric and suicidal behaviour outcomes at low, medium, and high average dosage

|  | **Dosage** | | |
| --- | --- | --- | --- |
|  | **Low**  **<0.5 DDDs/day** | **Medium**  **0.5-1.5 DDDs/day** | **High**  **>1.5 DDDs/day** |
| **Outcome** | **HR (95% CI)** | **HR (95% CI)** | **HR (95% CI)** |
| **Anxiety** | 1.19 (1.12, 1.26) | 1.38 (1.26, 1.51) | 1.40 (1.22, 1.61) |
| **Bipolar disorder** | 1.13 (1.01, 1.27) | 1.42 (1.17, 1.71) | 1.59 (1.18, 2.15) |
| **Depression** | 1.06 (0.97, 1.15) | 1.13 (0.97, 1.32) | 1.15 (0.92, 1.44) |
| **Psychotic disorder** | 1.16 (1.02, 1.31) | 1.25 (1.01, 1.54) | 1.06 (0.73, 1.56) |
| **Suicidal behaviour** | 1.03 (0.91, 1.17) | 1.12 (0.92, 1.38) | 1.09 (0.78, 1.51) |
|  | | | |

## Table S11. Psychiatric and suicidal behaviour outcomes in individuals dispensed a glucocorticoid (medication-only cohort) using a within-individual analysis in the data linkage covering 2006-2020, compared to the data linkage covering 2006-2013 only

|  | **Data linkage 2006-2020**  **(N= 1,105,964)** | **Data linkage 2006-2013**  **(N=680,215)** |
| --- | --- | --- |
|  | **HR (95% CI)** | **HR (95% CI)** |
| **Anxiety disorders** | 1.25 (1.20, 1.31) | 1.36 (1.25, 1.49) |
| **Bipolar disorders** | 1.23 (1.12, 1.36) | 1.37 (1.11, 1.71) |
| **Depressive disorders** | 1.08 (1.00, 1.16) | 1.06 (0.92, 1.23) |
| **Schizophrenia-spectrum disorders** | 1.17 (1.05, 1.30) | 0.93 (0.73, 1.20) |
| **Suicidal behaviour** | 1.06 (0.96, 1.17) | 1.11 (0.94, 1.31) |

## Table S12. Per-protocol analyses in individuals with an autoimmune disorder or gastrointestinal autoimmune disorder diagnosis (indication cohorts)

|  |  | **Autoimmune disorder cohort (N = 287,366)** | | | | **Gastrointestinal disorder cohort (N=96,029)** | | |
| --- | --- | --- | --- | --- | --- | --- | --- | --- |
|  |  | **Cumulative Incidence (%)** | |  |  | **Cumulative Incidence (%)** | |  |
| **Outcome** | **Time** | **Initiators** | **Controls** | **Hazard Ratio (95% CI)** | | **Initiators** | **Controls** | **Hazard Ratio (95% CI)** |
| **Anxiety** | **14 days** | 0.10 (0.05,0.15) | 0.06 (0.05,0.07) | 1.64 (0.94,2.86) | | 0.09 (0.01,0.16) | 0.06 (0.04,0.07) | 1.46 (0.61,3.51) |
|  | **120 days** | 0.53 (0.36,0.71) | 0.40 (0.36,0.43) | 1.47 (1.00,2.16) | | 0.43 (0.21,0.66) | 0.38 (0.32,0.45) | 1.26 (0.69,2.32) |
|  | **365 days** | 1.05 (0.57,1.54) | 0.97 (0.91,1.03) | 1.25 (0.90,1.76) | | 1.07 (0.17,1.96) | 0.90 (0.79,1.01) | 1.11 (0.64,1.95) |
| **Bipolar disorder** | **14 days** | 0.01 (0.00,0.03) | 0.01 (0.01,0.02) | 1.01 (0.27,3.76) | | 0.01 (0.00,0.04) | 0.01 (0.00,0.02) | 1.20 (0.07,19.95) |
|  | **120 days** | 0.12 (0.03,0.22) | 0.09 (0.07,0.10) | 1.24 (0.49,3.13) | | 0.11 (0.00,0.25) | 0.06 (0.03,0.09) | 1.62 (0.38,6.88) |
|  | **365 days** | 0.25 (0.01,0.49) | 0.18 (0.15,0.21) | 1.44 (0.68,3.05) | | 0.15 (0.00,0.33) | 0.12 (0.07,0.16) | 1.66 (0.52,5.34) |
| **Depression** | **14 days** | 0.03 (0.00,0.06) | 0.04 (0.03,0.04) | 0.84 (0.33,2.14) | | 0.02 (0.00,0.05) | 0.03 (0.02,0.05) | 0.51 (0.09,3.06) |
|  | **120 days** | 0.23 (0.11,0.34) | 0.24 (0.21,0.26) | 0.90 (0.47,1.73) | | 0.22 (0.04,0.39) | 0.23 (0.18,0.29) | 0.71 (0.27,1.88) |
|  | **365 days** | 0.73 (0.27,1.20) | 0.59 (0.54,0.64) | 1.04 (0.63,1.72) | | 0.49 (0.00,1.07) | 0.60 (0.51,0.70) | 0.88 (0.41,1.92) |
| **Schizophrenia spectrum disorders** | **14 days** | 0.02 (0.00,0.04) | 0.02 (0.01,0.02) | 0.99 (0.21,4.60) | | 0.01 (0.00,0.04) | 0.02 (0.00,0.03) | 0.62 (0.00,4934.89) |
|  | **120 days** | 0.12 (0.03,0.20) | 0.09 (0.07,0.11) | 1.17 (0.46,2.98) | | 0.06 (0.00,0.14) | 0.08 (0.05,0.11) | 0.70 (0.00,422.08) |
|  | **365 days** | 0.19 (0.03,0.35) | 0.19 (0.16,0.21) | 1.20 (0.58,2.50) | | 0.10 (0.00,0.25) | 0.16 (0.11,0.20) | 0.72 (0.04,13.27) |
| **Suicidal behaviour** | **14 days** | 0.01 (0.00,0.03) | 0.02 (0.01,0.02) | 0.74 (0.17,3.33) | | 0.01 (0.00,0.03) | 0.02 (0.01,0.03) | 0.61 (0.06,6.74) |
|  | **120 days** | 0.07 (0.01,0.14) | 0.12 (0.10,0.14) | 0.67 (0.21,2.10) | | 0.09 (0.00,0.19) | 0.12 (0.09,0.16) | 0.68 (0.17,2.60) |
|  | **365 days** | 0.37 (0.00,0.74) | 0.32 (0.28,0.36) | 0.71 (0.32,1.57) | | 0.23 (0.00,0.59) | 0.33 (0.26,0.40) | 0.72 (0.24,2.15) |

## Table S13. Intention-to-treat analyses in individuals with a gastrointestinal autoimmune disorder diagnosis (indication cohort)

|  |  | **Overall cohort (N = 96,029)** | | | **Past psychiatric diagnosis (N= 11,334)** | | |
| --- | --- | --- | --- | --- | --- | --- | --- |
|  |  | **Cumulative incidence (%)** | |  | **Cumulative incidence (%)** | |  |
| **Outcome** | **Time** | **Initiators** | **Controls** | **Hazard Ratio (95% CI)** | **Initiators** | **Controls** | **Hazard Ratio (95% CI)** |
| **Anxiety** | **14 days** | 0.08 (0.04,0.11) | 0.06 (0.05,0.07) | 1.31 (0.82,2.09) | 0.49 (0.23,0.75) | 0.34 (0.26,0.41) | 1.47 (0.86,2.51) |
|  | **120 days** | 0.47 (0.34,0.60) | 0.39 (0.35,0.43) | 1.26 (0.88,1.81) | 3.06 (2.16,3.97) | 2.16 (1.89,2.44) | 1.45 (0.97,2.16) |
|  | **365 days** | 1.06 (0.87,1.25) | 0.95 (0.88,1.01) | 1.18 (0.92,1.51) | 5.81 (4.59,7.03) | 4.86 (4.42,5.30) | 1.35 (1.03,1.76) |
| **Bipolar disorder** | **14 days** | 0.01 (0.00,0.03) | 0.01 (0.01,0.02) | 1.11 (0.28,4.39) | 0.08 (0.00,0.18) | 0.09 (0.05,0.13) | 0.83 (0.14,5.04) |
|  | **120 days** | 0.07 (0.02,0.12) | 0.06 (0.04,0.07) | 1.18 (0.41,3.42) | 0.46 (0.08,0.83) | 0.48 (0.35,0.61) | 0.89 (0.23,3.51) |
|  | **365 days** | 0.17 (0.09,0.25) | 0.12 (0.10,0.15) | 1.29 (0.65,2.53) | 1.15 (0.53,1.76) | 1.00 (0.81,1.19) | 1.00 (0.45,2.23) |
| **Depression** | **14 days** | 0.02 (0.01,0.03) | 0.03 (0.03,0.04) | 0.58 (0.29,1.19) | 0.11 (0.02,0.21) | 0.19 (0.14,0.24) | 0.61 (0.24,1.52) |
|  | **120 days** | 0.21 (0.13,0.28) | 0.24 (0.21,0.27) | 0.72 (0.42,1.24) | 1.09 (0.59,1.59) | 1.33 (1.12,1.54) | 0.71 (0.36,1.43) |
|  | **365 days** | 0.73 (0.56,0.89) | 0.63 (0.58,0.69) | 0.96 (0.69,1.33) | 3.30 (2.29,4.32) | 3.33 (2.96,3.69) | 0.88 (0.58,1.33) |
| **Schizophrenia spectrum disorders** | **14 days** | 0.01 (0.00,0.02) | 0.02 (0.01,0.02) | 0.66 (0.18,2.51) | 0.06 (0.00,0.15) | 0.12 (0.07,0.16) | 0.53 (0.09,3.16) |
|  | **120 days** | 0.07 (0.03,0.12) | 0.08 (0.06,0.10) | 0.79 (0.29,2.16) | 0.47 (0.12,0.83) | 0.63 (0.47,0.78) | 0.64 (0.17,2.39) |
|  | **365 days** | 0.15 (0.09,0.22) | 0.16 (0.14,0.19) | 0.93 (0.49,1.77) | 1.10 (0.55,1.65) | 1.21 (0.99,1.42) | 0.81 (0.37,1.75) |
| **Suicidal behaviour** | **14 days** | 0.01 (0.00,0.03) | 0.02 (0.01,0.02) | 0.82 (0.33,2.04) | 0.13 (0.03,0.23) | 0.10 (0.06,0.15) | 1.22 (0.49,3.06) |
|  | **120 days** | 0.11 (0.06,0.16) | 0.12 (0.10,0.14) | 0.87 (0.43,1.75) | 0.77 (0.38,1.16) | 0.68 (0.52,0.84) | 1.17 (0.58,2.37) |
|  | **365 days** | 0.36 (0.25,0.47) | 0.34 (0.30,0.38) | 0.95 (0.61,1.46) | 1.65 (0.96,2.34) | 1.67 (1.41,1.93) | 1.08 (0.66,1.76) |
